# Unveiling the underlying molecular mechanisms of high lutein production efficiency in *Chlorella sorokiniana* FZU60 under a mixotrophy/photoautotrophy two-stage strategy by transcriptomic, physiological, and biochemical analyses

**DOI:** 10.1186/s13068-023-02300-8

**Published:** 2023-03-15

**Authors:** Ruijuan Ma, Zhen Zhang, Hong Fang, Xinyu Liu, Shih-Hsin Ho, Youping Xie, Jianfeng Chen

**Affiliations:** 1 Marine Biological Manufacturing Center of Fuzhou Institute of Oceanography, Fuzhou University, Fuzhou, 350108 China; 2 Technical Innovation Service Platform for High Value and High-Quality Utilization of Marine Organism, Fuzhou University, Fuzhou, 350108 China; 3 Fujian Engineering and Technology Research Center for Comprehensive Utilization of Marine Products Waste, Fuzhou University, Fuzhou, 350108 China; 4 Fuzhou Industrial Technology Innovation Center for High-Value Utilization of Marine Products, Fuzhou University, Fuzhou, 350108 China; 5 State Key Laboratory of Urban Water Resource and Environment, School of Environment, Harbin Institute of Technology, Harbin, 150090 China

**Keywords:** *Chlorella sorokiniana* FZU60, Lutein, Mixotrophy, Photoautotrophy, Molecular mechanisms

## Abstract

**Background:**

*Chlorella sorokiniana* FZU60 is a promising lutein producing microalga. A mixotrophy/photoautotrophy two-stage strategy can achieve high biomass concentration at stage 1 and high lutein content at stage 2, leading to excellent lutein production efficiency in *C. sorokiniana* FZU60. However, the underlying molecular mechanisms are still unclear, restraining the further improvement of lutein production.

**Results:**

In this study, physiological and biochemical analysis revealed that photochemical parameters (Fv/Fm and NPQ) and photosynthetic pigments contents increased during the shift from mixotrophy to photoautotrophy, indicating that photosynthesis and photoprotection enhanced. Furthermore, transcriptomic analysis revealed that the glyoxylate cycle and TCA cycle were suppressed after the shift to photoautotrophy, leading to a decreased cell growth rate. However, the gene expression levels of photosynthesis, CO_2_ fixation, autophagy, and lutein biosynthesis were upregulated at the photoautotrophy stage, demonstrating that microalgal cells could obtain more precursor to synthesize lutein for enhancing photosynthesis and reducing reactive oxygen species.

**Conclusions:**

The findings help to elucidate the molecular mechanisms for high lutein production efficiency of *C. sorokiniana* FZU60 under the mixotrophy/photoautotrophy strategy, identify key functional genes responsible for lutein biosynthesis, and shed light on further improvement of lutein production by genetic or metabolic engineering in future studies.

**Supplementary Information:**

The online version contains supplementary material available at 10.1186/s13068-023-02300-8.

## Introduction

Lutein, a primary xanthophyll carotenoid, has many beneficial effects on human health, such as protection of ocular health, anti-inflammatory, beneficial effects in the development of infant brain, and inhibition of adipogenesis [1]. Thus, it has been widely used in food additives, cosmetics, and drugs [2]. The lutein market was valued at USD 135 million in 2015 and is expected to have an annual growth rate of 6% by 2024 [1]. Marigold flowers are the traditional lutein source, while lutein production from them has the disadvantages of high labor intensity, low lutein content, occupation of arable land, and susceptible to climate [3]. In recent years, microalgae have been considered an alternative lutein source due to the advantages of a fast growth rate, high lutein production, and independence of arable land and fresh water resources [2, 4].

The production of microalgae-based lutein can be manipulated in photoautotrophic, mixotrophic, and heterotrophic modes [1, 5]. Among them, photoautotrophy and mixotrophy involve CO_2_ fixation [6], and thus are relatively cost-effective if flue gas is used as the CO_2_ source [7]. To date, photoautotrophy is the most widely used cultivation mode for microalgae as it is easy to operate and enables the utilization of freely available sunlight [8]. Besides, the biosynthesis of light-induced lutein enhances under this cultivation mode [9]. However, photoautotrophy is limited by light penetration due to the self-shading effects of microalgal cells when cell density increases, leading to a low biomass production [10]. Microalgae cultivated in mixotrophic mode can use both inorganic and organic carbon sources for photosynthesis and aerobic respiration; therefore, the cell growth rate is much higher than that of photoautotrophic mode [11]. However, lutein biosynthesis reduces under the mixotrophic mode [12]. Based on these phenomena, a two-stage strategy with semi-batch mixotrophic cultivation in stage 1 and photoautotrophic induction in stage 2 was explored to initially improve cell growth and then induce lutein accumulation in *Chlorella sorokiniana* MB-1, achieving a high lutein productivity of 7.62 mg/L/d [13]. Likewise, a multi-operation integrated strategy with semi-batch and fed-batch mixotrophic cultivation in stage 1 and photoinduction in stage 2 was applied in *C. sorokiniana* FZU60 to achieve an excellent lutein content, productivity, and production of 9.57 mg/g, 11.57 mg/L/d, and 17.35 mg/L, respectively [9]. Moreover, the mixotrophy/photoautotrophy two-stage strategy could be scaled in a 50 L column photobioreactor in *C. sorokiniana* FZU60 [12]. Nevertheless, the molecular mechanisms for high lutein production efficiency under the mixotrophy/photoautotrophy two-stage strategy have not been elucidated.

Microalgal lutein is connected to light-harvesting complexes (LHCs), presented as a “structural form” for light-harvesting; besides, it functions to dissipate excess light energy for protecting microalgae from photo-oxidative damage by non-photochemical quenching [14, 15]. Hence, the accumulation of lutein is highly associated with photosynthesis. It was found that photosynthesis was shut off by reducing the expression of photosynthetic apparatus protein, including core proteins D2 and CP43 of photosystem II (PSII), core protein PsaA of photosystem I (PSI), and large subunit cytochrome *b*_6_ of the cytochrome *b*_6_*f* (Cyt* b*_6_*f*) complex, when the microalga *Chromochloris zofingiensis* was transferred from photoautotrophic to mixotrophic cultivation [16]. In addition, the transcriptional level analysis showed that non-photochemical quenching and photorespiration of *C. zofingiensis* were significantly decreased under mixotrophic condition, compared with that under photoautotrophic condition [17]. Thus, the shift from mixotrophy to photoautotrophy may enhance photosynthesis and photoprotection, resulting in an increase in lutein accumulation due to its functions in light-harvesting and non-photochemical quenching. However, the underlying molecular mechanisms need to be studied.

The present study investigated the growth, physiological, and biochemical parameters of *C. sorokiniana* FZU60 under the mixotrophy/photoautotrophy two-stage strategy. Furthermore, transcriptomic analysis was used to reveal the features of photosynthesis, carbon fixation, autophagy, and lutein biosynthesis under this trophic transition. The findings shed light on the molecular mechanisms for high lutein production efficiency of *C. sorokiniana* FZU60 under the mixotrophy/photoautotrophy strategy and will provide a foundation for future studies on further improvement of lutein production by genetic or metabolic engineering.

## Results and discussion

### Changes in growth, lutein accumulation, and photochemical parameters under the mixotrophy/photoautotrophy two-stage strategy

**Fig. 1:**
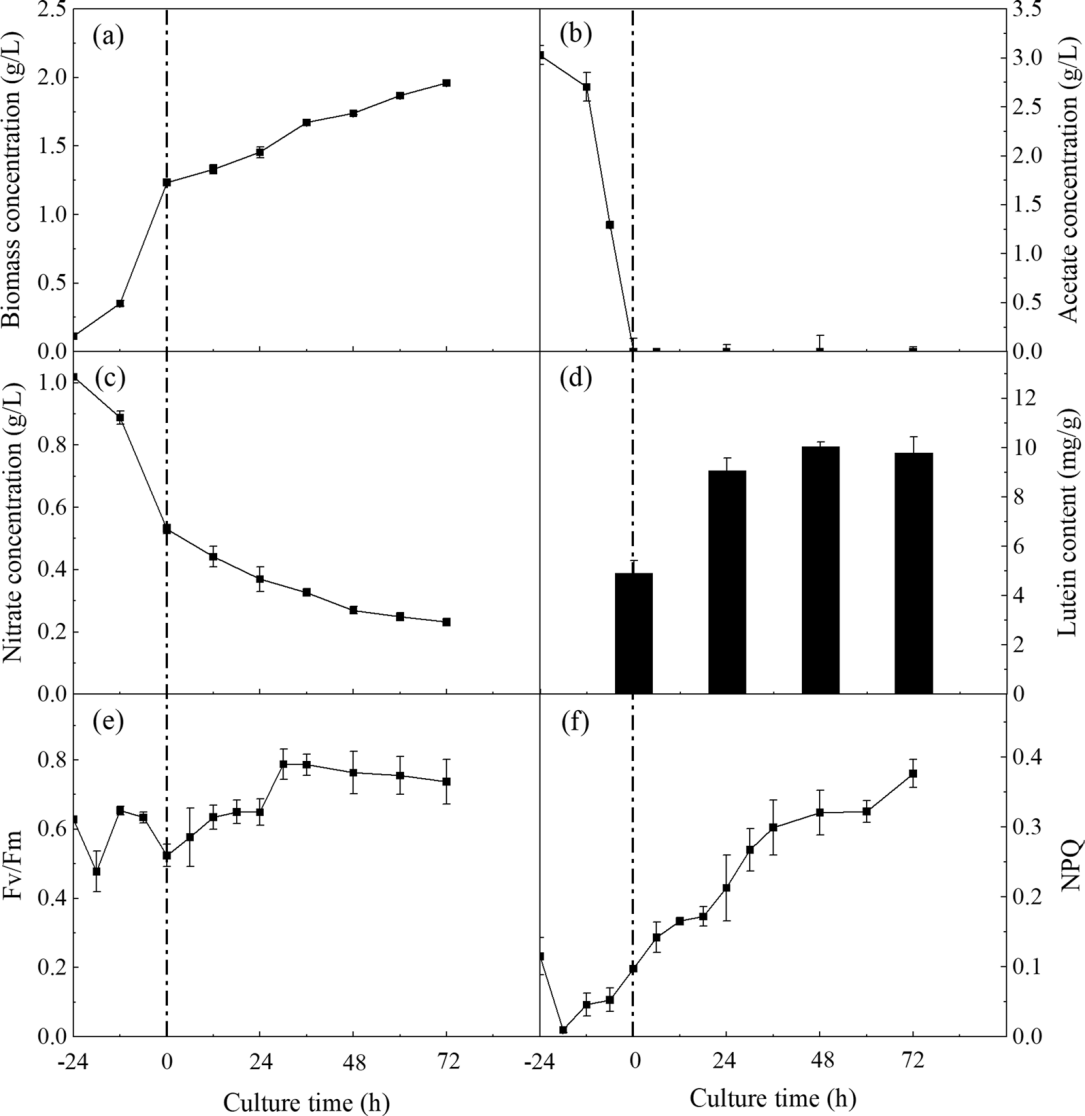
Time-course profiles of growth, lutein accumulation, and photochemical parameters of *C. sorokiniana* FZU60 under the mixotrophy/photoautotrophy strategy. **a** Biomass concentration; **b** Acetate concentration; **c** Nitrate concentration; **d** Lutein content; **e** Fv/Fm; **f** NPQ. The culture time at the onset of acetate depletion was denoted as 0 h

As shown in Fig. 1a, biomass concentration raised rapidly from -24 to 0 h, when acetate was replete (Fig. 1b). Then, the growth rate decreased after acetate was depleted (0–72 h). Consistently, nitrate was consumed quickly from -24 to 0 h, and then the consumption rate decreased from 0 to 72 h (Fig. 1c). Hence, microalgal cells grew faster under mixotrophic condition, compared with that under photoautotrophic condition. This result is similar to the studies in *C. zofingiensis*[16], *C. sorokiniana* MB-1 [13], and *Scenedesmus obliquus* KGE‑17 [18]. The higher growth rate under mixotrophic condition might be due to the fact that microalgal cells could simultaneously utilize inorganic and organic carbon sources under lighting condition for photosynthesis and aerobic respiration [11].

On the other hand, lutein content raised significantly from 0 to 48 h, and then plateaued at 72 h (Fig. 1d), indicating that photoautotrophy can stimulate lutein accumulation. Lutein accumulation is highly associated with photosynthesis due to its function in light-harvesting and photoprotection [14, 15]. Hence, the photochemical parameters of Fv/Fm and NPQ representing photosynthesis performance were analyzed. As shown in Fig. 1e, the value of Fv/Fm fluctuated from -24 to 0 h, and then gradually increased from 0 to 30 h. The increase in Fv/Fm value during the shift from mixotrophic to photoautotrophic conditions indicated that the photosynthetic capacity improved [19]. Besides, the value of NPQ decreased initially, followed by a steady increase under mixotrophic condition, and then continuously increased after the transfer to photoautotrophic condition (Fig. 1f). The sharp increase in NPQ value under photoautotrophic condition revealed that the dissipation of light energy increased, which could be a result of the microalgal photoprotection mechanism [20]. Hence, the shift from photoautotrophy to mixotrophy improved photosynthetic capacity and photoprotection, leading to an increase in lutein accumulation.

### Variations of biochemical and pigmental compositions under the mixotrophy/photoautotrophy two-stage strategy

**Fig. 2:**
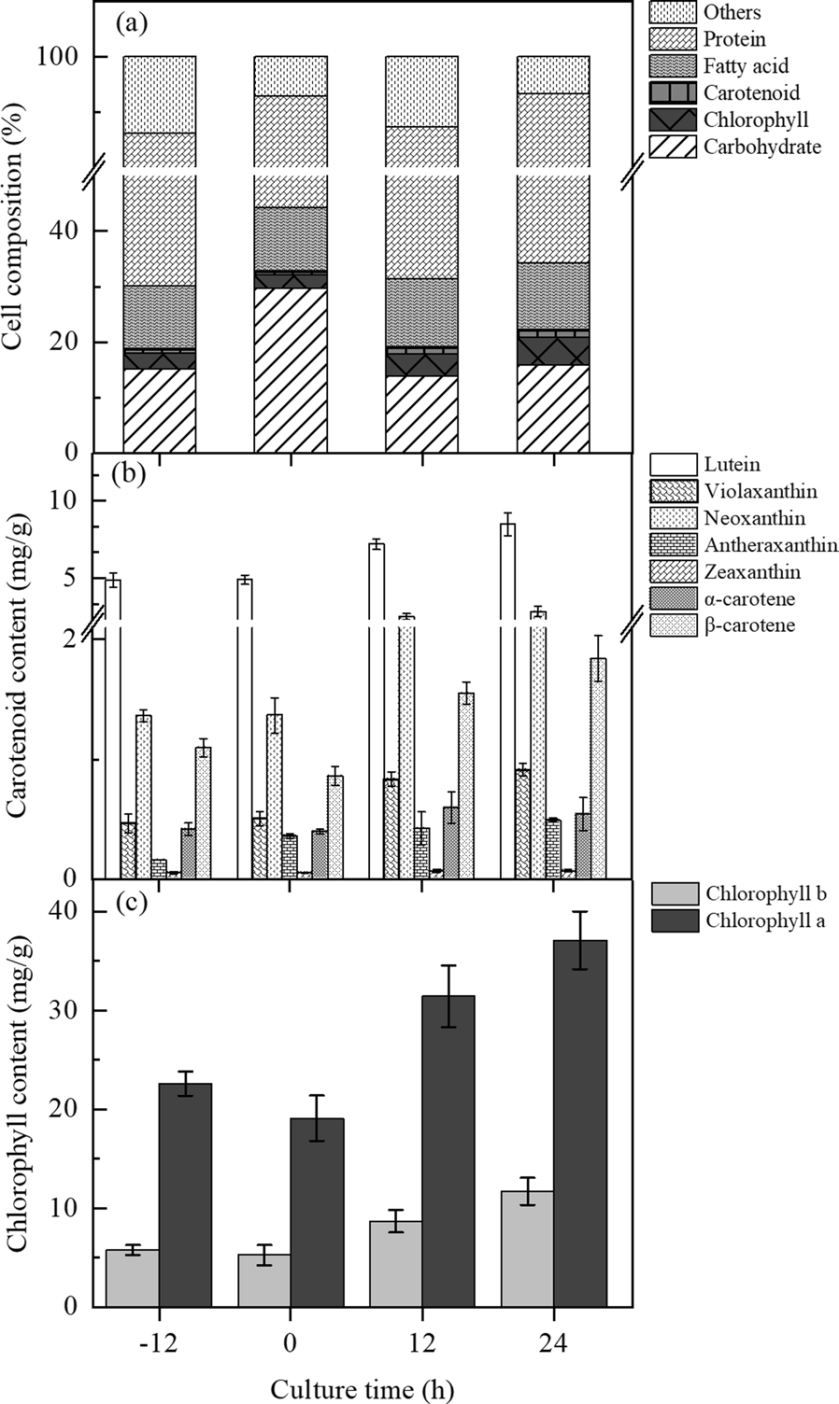
Time-course profiles of biochemical (**a**), carotenoid (**b**), and chlorophyll (**c**) compositions of *C. sorokiniana* FZU60 under the mixotrophy/photoautotrophy strategy. The culture time at the onset of acetate depletion was denoted as 0 h

Biochemical compositions reveal the physical metabolism of microalgal cells. Thus, the variations in biochemical compositions of *C. sorokiniana* FZU60 under the mixotrophy/photoautotrophy strategy were investigated. As shown in Fig. 2a, the cellular composition consisted mainly of protein, carbohydrate, fatty acid, carotenoid, and chlorophyll. Protein was the major component of microalgal cells. Its content decreased at 0 h, and then increased at 12 and 24 h. The changing trend of carbohydrate content was opposite to protein content, which increased at 0 h, and then declined at 12 and 24 h. No significant difference was observed in fatty acid content. Protein is a primary metabolite, which is accumulated under the optimal conditions for cell growth, while carbohydrate is classified into structural and storage types, the latter of which (such as starch) is largely accumulated under stressed conditions as short-term energy reserve [21]. An increase in carbohydrate content, especially starch content, under stressed conditions has been observed in many microalgae, such as *Chlorella* species [22] and *Neochloris oleoabundans* HK-129 [23]. Thus, the sudden increase in carbohydrate content and decrease in protein content at the onset of acetate depletion indicated that the shift from mixotrophy to photoautotrophy might course transient stress to microalgal cells. Subsequently, carbohydrate content decreased, and protein content increased, when microalgal cells adjusted to the photoautotrophic condition.

The contents of pigments (including carotenoid and chlorophyll) were not significantly different from − 12 to 0 h, while their contents increased sharply from 0 to 24 h (Fig. 2a). It can be observed explicitly in Fig. 2b that the contents of carotenoids, including lutein, violaxanthin, neoxanthin, antheraxanthin, zeaxanthin, α-carotene, and β-carotene, significantly enhanced after microalgal cells were shifted to photoautotrophic condition. Similarly, the contents of chlorophylls, including chlorophyll a and b, significantly increased under photoautotrophic condition (Fig. 2c). Both carotenoid and chlorophyll are photosynthetic pigments [24]. The capture of light by PSII was achieved by a macromolecular complex consisting of major pigments (chlorophyll a and b) and minor pigments (carotenoids of lutein, neoxanthin, and violaxanthin) [25]. The significant increase in their contents indicated that photosynthesis might be greatly enhanced during the shift from mixotrophy to photoautotrophy.

### Global gene response at the transcriptional level during the shift from mixotrophy to photoautotrophy

**Fig. 3:**
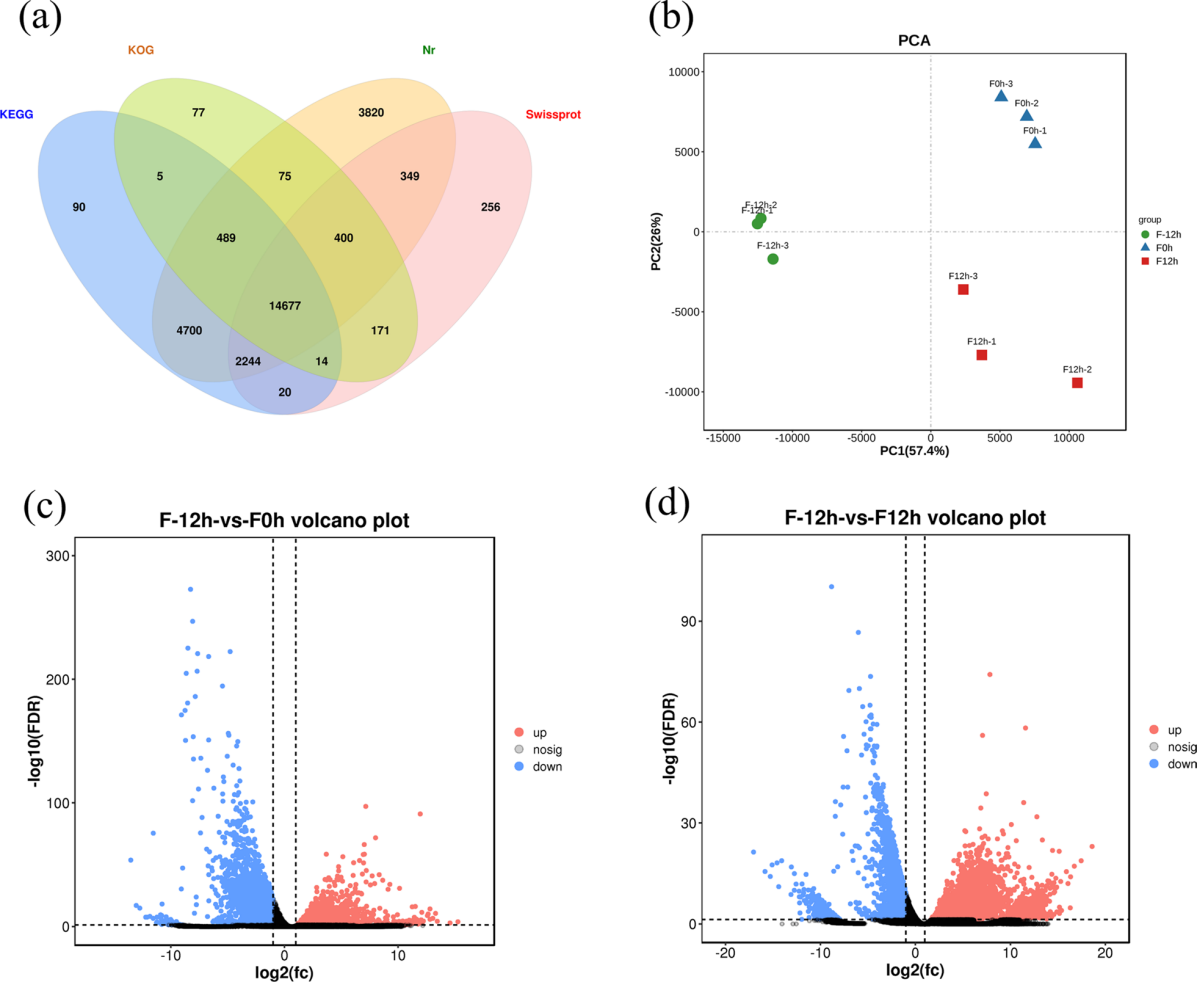
Global analysis of transcriptomes and DEGs. **a** Venn diagram of annotation results in four databases; **b** Score plot of principle component analysis; **c** Volcano plot of changes in gene expression between F-12 h and F0h groups; **d** Volcano plot of changes in gene expression between F-12 h and F12h groups

To further investigate the underlying molecular mechanisms of high lutein production efficiency in *C. sorokiniana* FZU60 under the mixotrophy/photoautotrophy two-stage strategy, a transcriptomic analysis was performed based on de novo assembly methods. The obtained unigenes mainly distributed between 150 and 1000 bp (Additional file 1: Fig. S1a). The annotation results indicated that there were 14,677 unigenes annotated in all four databases of KEGG, KOG, Nr, and Swissprot (Fig. 3a). According to the results of Nr annotation, the unigenes were most aligned to *C. sorokiniana* (10,616 unigenes) (Additional file 1: Fig. S1b), confirming that the newly isolated microalga is a species of *C. sorokiniana*. The principal component analysis demonstrated that all transcriptomes were highly corelated with each other within each group (Fig. 3b). DEGs analysis showed that the number of DEGs was distinct between treatment groups (Additional file 1: Fig. S2). Compared with F-12 h group, there was 2323 unigenes were upregulated and 2254 unigenes were downregulated for F0h group (Fig. 3c and Additional file 1: Fig. S2); besides, 12,267 unigenes were upregulated and 2287 unigenes were downregulated for F12h group (Fig. 3d and Additional file 1: Fig. S2). Moreover, the unigenes involved in the pathways of acetate metabolism, photosynthesis, CO_2_ fixation, autophagy, and carotenoid biosynthesis were manually identified and their dynamic changes in transcriptional levels were analyzed.

#### Suppression of glyoxylate cycle and TCA cycle during the shift from mixotrophy to photoautotrophy

**Fig. 4:**
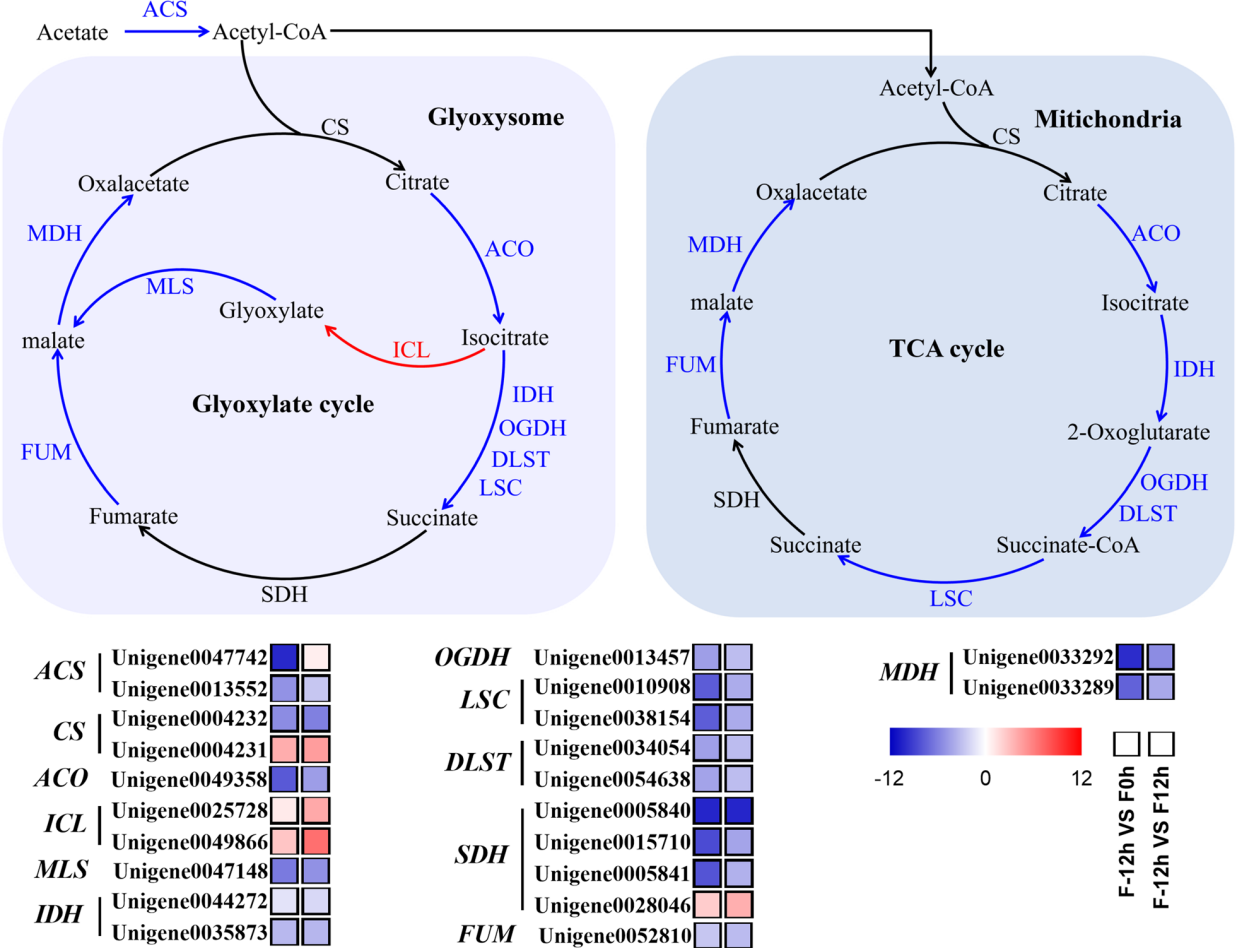
Transcriptional response of glyoxylate cycle and TCA cycle of *C. sorokiniana* FZU60 under the mixotrophy/photoautotrophy strategy. ACO: aconitate hydratase; ACS: acetyl-CoA synthetase; CS: citrate synthase; DLST: dihydrolipoamide succinyltransferase; FUM: fumarate hydratase; ICL: isocitrate lyase; IDH: isocitrate dehydrogenase; LSC: succinyl-CoA synthetase; MDH: malate dehydrogenase; MLS: malate synthase; OGDH: 2-oxoglutarate dehydrogenase; SDH: succinate dehydrogenase. The red, blue, and black arrows indicate the upregulation, downregulation, and invariability of gene, respectively

Acetate is the organic carbon source used by microalgal cells at the mixotrophy stage, and it was exhausted at the photoautotrophy stage. Thus, the metabolism of acetate might be changed completely during the shift from mixotrophy to photoautotrophy. After uptake in microalgae, acetate is converted into acetyl-CoA at the action of ACS [26]. Results of the transcriptomic analysis showed that the expression level of *ACS* gene was significantly downregulated after acetate was depleted (Fig. 4), indicating that acetate metabolism was sharply suppressed. This result is consistent with the findings in *Chlamydomonas reinhardtii* that the expression level of *ACS* gene was significantly upregulated when microalgal cells were shifted from autotrophic to mixotrophic conditions [27].

Acetate can enter the glyoxylate cycle or TCA cycle, which exists in glyoxysome and mitochondria, respectively [28, 29]. The glyoxylate cycle and TCA cycle share similar enzymes, except for two special enzymes (MLS and ICL) in the glyoxylate cycle [28]. As shown in Fig. 4, the expression levels of *ACO*, *IDH*, *OGDH*, *DLST*, *LSC*, *FUM*, and *MDH* genes, presenting in both the glyoxylate cycle and TCA cycle, were significantly downregulated. Besides, the expression levels of *ICL* and *MLS* genes, existing in the glyoxylate cycle, were upregulated and downregulated, respectively. Hence, the transcriptomic analysis showed that most genes in the glyoxylate cycle and TCA cycle were downregulated, indicating that these two pathways were suppressed. The glyoxylate cycle or TCA cycle can provide carbon skeletons and energy (ATP and NADPH) for microalgal cells, which are important for cell growth [30]. Hence, the decrease in cell growth rate during the shift from mixotrophy to photoautotrophy (Fig. 1a) could be due to the suppression of glyoxylate cycle and TCA cycle.

#### Enhancement of photosynthesis and CO_2_ fixation during the shift from mixotrophy to photoautotrophy

**Fig. 5:**
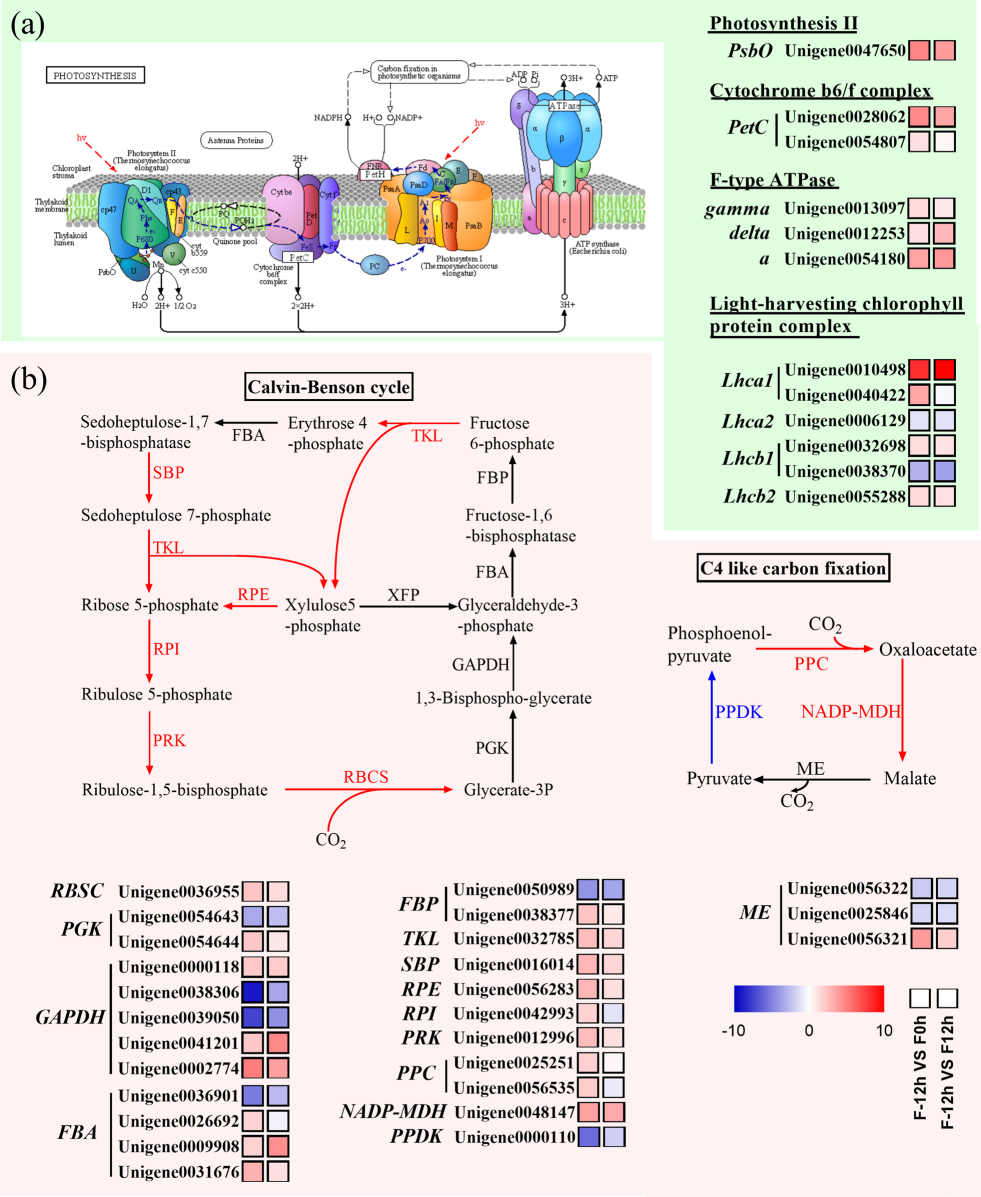
Transcriptional response of photosynthesis **a** and CO_2_ fixation **b** of *C. sorokiniana* FZU60 under the mixotrophy/photoautotrophy strategy. a: F-type H^+^-transporting ATPase subunit a; delta: F-type H + -transporting ATPase subunit delta; FBA: fructose-bisphosphate aldolase; FBP: fructose-1,6-bisphosphatase I; gamma: F-type H^+^-transporting ATPase subunit gamma; GAPDH: glyceraldehyde 3-phosphate dehydrogenase; Lhca1: light-harvesting complex I chlorophyll a/b binding protein 1; Lhca2: light-harvesting complex I chlorophyll a/b binding protein 2; Lhcb1: light-harvesting complex II chlorophyll a/b binding protein 1; Lhcb2: light-harvesting complex II chlorophyll a/b binding protein 2; ME: malic enzyme; NADP-MDH: chloroplast NADP-malate dehydrogenase; PetC: cytochrome *b*_6_*f* complex iron-sulfur subunit; PGK: phosphoglycerate kinase; PPC: phosphoenolpyruvate carboxylase; PPDK: pyruvate orthophosphate dikinase; PRK: phosphoribulokinase; PsbO: photosystem II oxygen-evolving enhancer protein 1; RBCS: ribulose-bisphosphate carboxylase; RPE: ribulose-phosphate 3-epimerase; RPI: ribose 5-phosphate isomerase; SBP: sedoheptulose-1,7-bisphosphatase; TKL: transketolase. The red, blue, and black arrows indicate the upregulation, downregulation, and invariability of gene, respectively

Since photosynthesis and CO_2_ fixation are closely related to lutein biosynthesis, the changes of them in transcriptomic level were investigated in this study. Photosynthetic apparatus mainly consists of five complexes, including PS I, PS II, Cyt* b*_6_*f* complex, photosynthetic electron transport, and F-type ATPase [31]. As shown in Fig. 5a, the expression levels of *PsbO* gene in PSII, *PetC* gene in Cyt* b*_6_*f* complex, as well as *gamma*, *delta*, and *a* genes in F-type ATPase were significantly upregulated at 0 and 12 h. In addition, the expression of *Lhca1*, *Lhcb1* (Unigene0032698), and *Lhcb2* genes in LHCs were upregulated after the shift to photoautotrophy. Lutein is combined with LHCs and functions in light-harvesting and photoprotection [14, 15]. The enhanced gene expression of PSII and LHCs indicated that photosynthesis increased, which might require more lutein and thus stimulated lutein accumulation. Besides, F-type ATPase is responsible for the generation of energy molecule ATP using H^+^ produced by the PS II and Cyt* b*_6_*f* complex [32]. The increase in gene expression of the F-type ATPase and Cyt* b*_6_*f* complex indicated that the ATP synthesis enhanced, which could be used as energy for CO_2_ fixation and carotenoid accumulation. Hence, the upregulation of genes coding photosynthetic apparatus was in line with the increase in Fv/Fm value and the content of photosynthetic pigments.

Similar to some other microalgae, such as *Chromochloris zofingiensis* [31] and *Thalassiosira weissflogii* [33], both the C_4_ cycle and Calvin–Benson cycle for CO_2_ fixation exist in *C. sorokiniana* FZU60. As shown in Fig. 5b, the expression level of *RBCS* gene in the Calvin–Benson cycle, responsible for fixing CO_2_ into glycerate, was significantly upregulated after the shift to photoautotrophy (0 and 12 h). Besides, the expression levels of *SBP*, *TKL*, *RPI*, *PRK*, and *RPE* genes were all upregulated at 0 and 12 h. Meanwhile, the expression level of some isoforms of *GAPDH*, *PGK*, *FBA*, and *FBP* was upregulated at 0 and 12 h. Thus, CO_2_ fixation in the Calvin–Benson cycle was significantly enhanced. Glyceraldehyde-3-phosphate, an essential product of the Calvin–Benson cycle, can be converted into pyruvate, which is the substrate for biosynthesizing the precursor (IPP) of carotenoid [21, 31]. Therefore, the enhanced Calvin–Benson cycle might help to provide more precursor for carotenoid accumulation.

On the other hand, the expression level of *ppc* gene in the C_4_ cycle pathway, responsible for the fixation of CO_2_ to oxaloacetate, was upregulated at 0 h (Fig. 5b). Besides, the expression level of *NADP-MDH* gene was significantly enhanced after the switch to photoautotrophy (0 and 12 h). The NADP-MDH catalyzes oxaloacetate into malate, which is further transformed into pyruvate, the initial metabolite of the MEP pathway for biosynthesizing the precursor (IPP) of carotenoid [21, 31]. Besides, the expression level of *PPDK* gene, responsible for catalyzing pyruvate into phosphoenol-pyruvate, was downregulated after the shift to photoautotrophy (0 and 12 h). Hence, the biosynthesis of pyruvate from oxaloacetate and malate increased, while the transformation of pyruvate into phosphoenol-pyruvate decreased, which could facilitate pyruvate accumulation and thus enhance carotenoid biosynthesis.

#### Enhancement of autophagy during the shift from mixotrophy to photoautotrophy

Autophagy is the main degradation pathway for recycling cellular waste components in microalgal cells, which is activated under stressed conditions, such as oxidative stress [34, 35]. It has been found that carotenoid biosynthesis and autophagy genesis are induced simultaneously to reduce reactive oxygen species (ROS), thus providing a defense against photo-oxidative damage [36]. The increase in NPQ value after the shift to photoautotrophy (Fig. 1f) indicated that the dissipation of light energy increased, which was used for the defense against photo-oxidative damage [20]. Hence, the shift from mixotrophy to photoautotrophy might result in an increase in ROS level and photo-oxidative damage to microalgal cells, thus enhancing carotenoid biosynthesis and autophagy genesis.

**Fig. 6:**
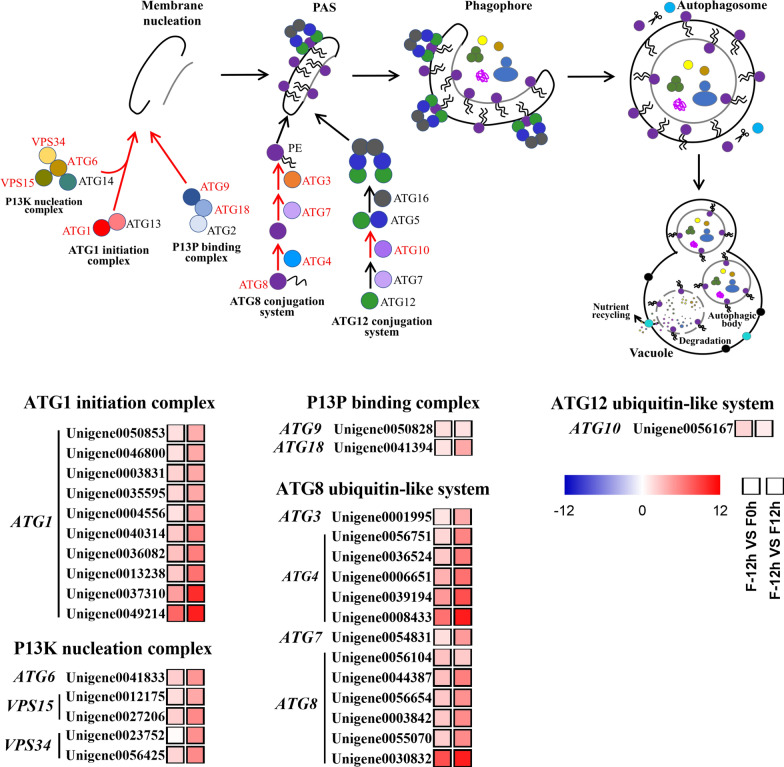
Transcriptional response of autophagy of *C. sorokiniana FZU60* under the mixotrophy/photoautotrophy strategy. ATG1: autophagy-related protein 1; ATG2: autophagy-related protein 2; ATG3: autophagy-related protein 3; ATG4: autophagy-related protein 4; ATG5: autophagy-related protein 5; ATG6: autophagy-related protein 6; ATG7: autophagy-related protein 7; ATG8: autophagy-related protein 8; ATG9: autophagy-related protein 9; ATG10: autophagy-related protein 10; ATG12: autophagy-related protein 12; ATG13: autophagy-related protein 13; ATG18: autophagy-related protein 18; VPS15: phosphoinositide-3-kinase; VPS34: phosphatidylinositol 3-kinase. The red, blue, and black arrows indicate the upregulation, downregulation, and invariability of gene, respectively

The autophagy machinery consists mainly of the ATG1 initiation complex, PI3K nucleation complex, PI3P binding complex, ATG8 ubiquitin-like system, and ATG12 ubiquitin-like system [37]. As shown in Fig. 6, the expression levels of *ATG1* gene in the ATG1 initiation complex, *ATG6*, *VPS15*, and *VPS34* genes in the PI3K nucleation complex, *ATG9* and *ATG18* genes in the PI3P binding complex, *ATG3*, *ATG4*, *ATG7*, and *ATG8* genes in the ATG8 ubiquitin-like system, as well as *ATG10* gene in the ATG12 ubiquitin-like system were all upregulated after the shift to photoautotrophy (0 and 12 h). To be noted, ATG8 protein is vital for the formation and maturation of autophagosome, a double membrane vesicle that engulfs cytosolic components [38]. The results showed that all five isoforms of *ATG8* gene were significantly upregulated. Thus, autophagy was strongly activated in *C. sorokiniana* FZU60 after the shift to photoautotrophy, indicating that ROS level might increase in microalgal cells, which could induce carotenoid biosynthesis simultaneously [36].

#### Enhancement of lutein biosynthesis during the shift from mixotrophy to photoautotrophy

**Fig. 7:**
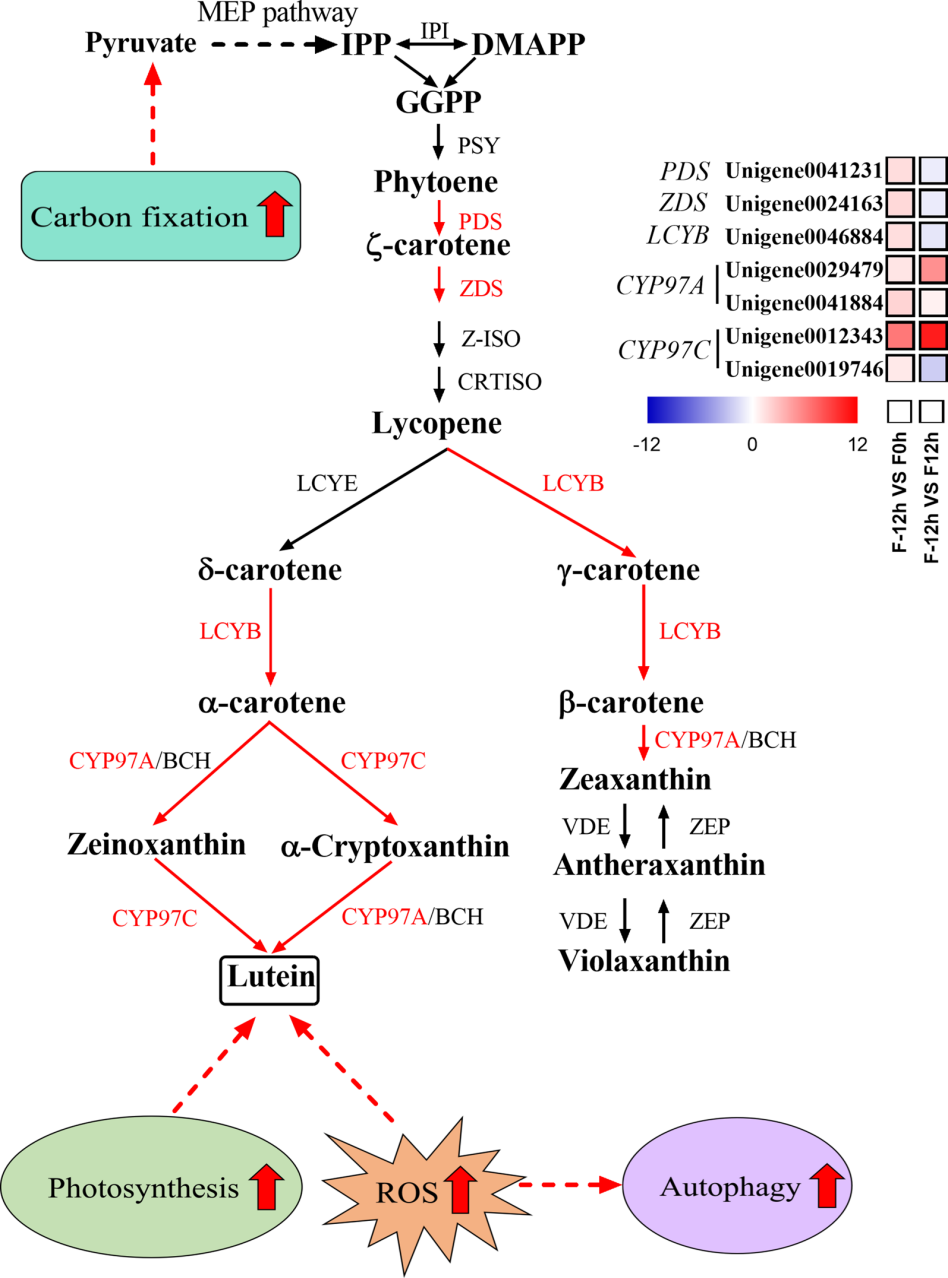
Transcriptional response of carotenoid biosynthesis of *C. sorokiniana FZU60* under the mixotrophy/photoautotrophy strategy. BCH: β-carotene hydroxylase; CRTISO: prolycopene isomerase; CYP97A: cytochrome P450 carotenoid hydroxylase A; CYP97C: cytochrome P450 carotenoid hydroxylase C; DMAPP: dimethylallyl diphosphate; IPP: isopentenyl diphosphate; LCYB: lycopene beta-cyclase; LCYE: lycopene epsilon-cyclase; PDS: phytoene desaturase; PSY: phytoene synthase; VDE: violaxanthin deepoxidase; ZDS: zeta-carotene desaturase; ZEP: zeaxanthin epoxidase; Z-ISO: zeta-carotene isomerase. The red, blue, and black arrows indicate the upregulation, downregulation, and invariability of gene, respectively

Lutein biosynthesis initiates from IPP and its isomer DMAPP, which are biosynthesized by the MEP pathway [1]. The product GGPP is converted into phytoene at the catalyzation of PSY [39]. Phytoene is transformed into ζ-carotene by PDS and then lycopene by ZDS, Z-ISO, and CRTISO [40]. As shown in Fig. 7, the expression levels of *PDS* and *ZDS* genes were significantly upregulated when acetate was depleted (0 h), which could lead to an enhanced accumulation of lycopene, a precursor of carotenoid, thus enhancing carotenoid biosynthesis (Fig. 2a). Studies in *Haematococcus pluvialis* indicated that the expression level of *PDS* gene was upregulated during carotenoid accumulation [41], and the overexpression of endogenous *PDS* gene significantly enhanced carotenoid accumulation [42].

Afterward, lycopene subsequently flows into two branches. For one branch, lycopene is catalyzed into δ-carotene and then α-carotene at the action of LCYE and LCYB [43]. Further, α-carotene is transformed into lutein through zeinoxanthin or α-cryptoxanthin at the action of CYP97C and BCH or CYP97A [44]. Results showed that *LCYB* gene was significantly upregulated at 0 h (Fig. 7), which was consistent with the increase in the contents of α-carotene and β-carotene (Fig. 2b). It should be noted that the expression levels of two isoforms of *CYP97A* gene and one isoform of *CYP97C* gene were upregulated at both 0 and 12 h (Fig. 7). CYP97A and CYP97C are important for catalyzing lutein biosynthesis [44]. Hence, the upregulation of *CYP97A* and *CYP97C* genes resulted in an increase in lutein content. For the other branch, lycopene is catalyzed into γ-carotene and then β-carotene by LCYB. Subsequently, β-carotene is converted into zeaxanthin by BCH or CYP97A [44], which is then transformed into antheraxanthin and violaxanthin, consisting of the violaxanthin cycle [45]. The enhanced contents of carotenoids in the violaxanthin cycle, including zeaxanthin, antheraxanthin, and violaxanthin (Fig. 2b), might be due to the upregulation of *LCYB* and *CYP97A* genes (Fig. 7).

Noticeably, the expression levels of *PDS*, *ZDS*, *LCYB* genes, and one isoform of *CYP97C* gene (Unigene0019746) were downregulated at 12 h. It has been found that gene expression generally precedes the biosynthesis of metabolites [31, 46]. Microalgal cells were under photoautotrophic condition from 0 h. Therefore, the upregulation of lutein biosynthesis genes at 0 h might lead to the translation of sufficient enzymes for enhanced lutein accumulation.

### Validation of selected genes by qPCR

**Fig. 8:**
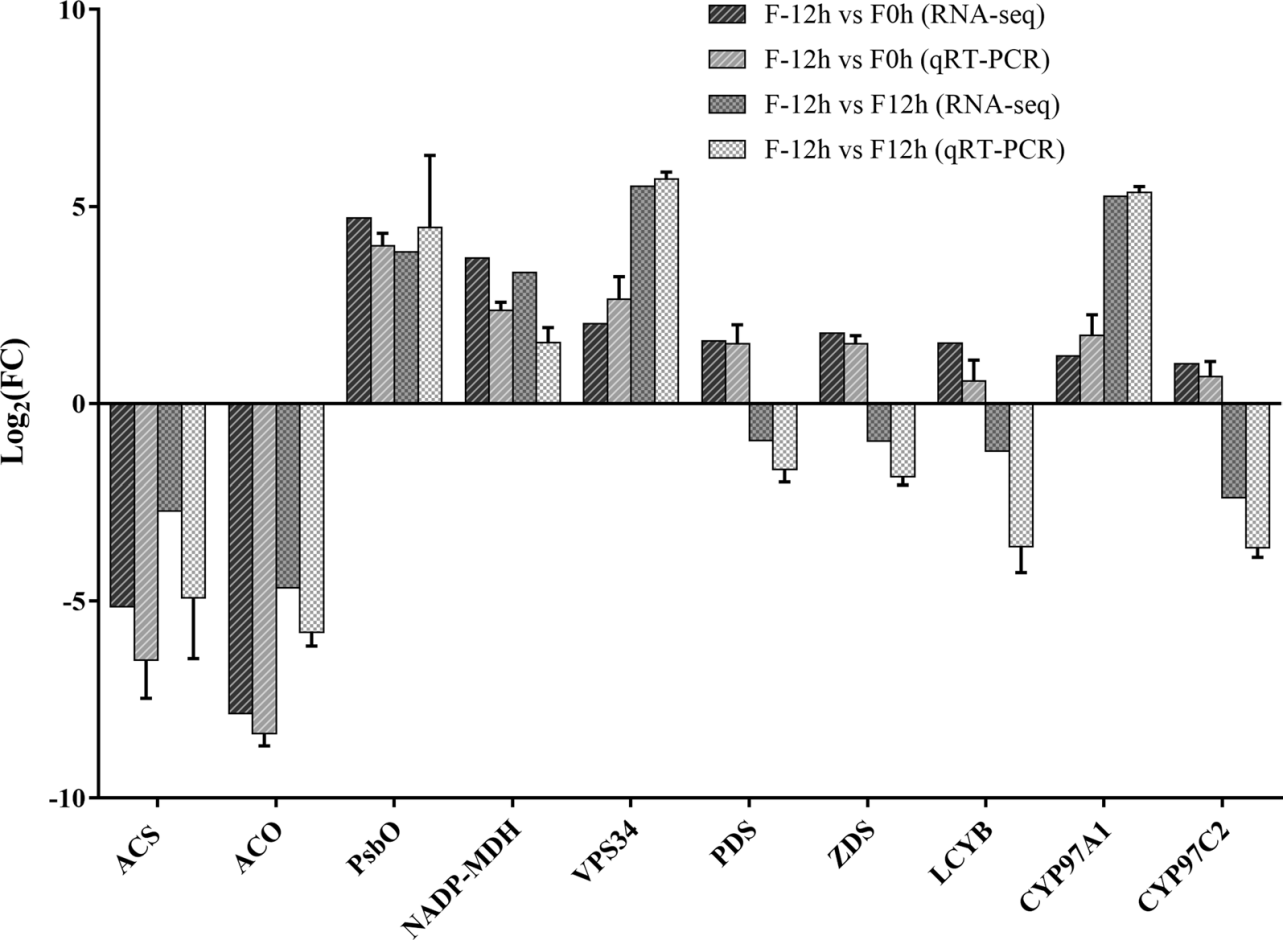
Expression validation of selected genes by qPCR. The unigene number of selected genes is as follows: *ACS* (Unigene0013552), *ACO* (Unigene0049358), *PsbO* (Unigene0047650), *NADP-MDH* (Unigene0047650), *VPS34* (Unigene0056425), *PDS* (Unigene0041231), *ZDS* (Unigene0024163), *LCYB* (Unigene0046884), *CYP97A* (Unigene0029479), and *CYP97C* (Unigene0019746)

To validate RNA-seq data, 10 genes were selected to analyze the expression pattern by qPCR. As shown in Fig. 8, the expression levels of *ACS* and *ACO* genes, involved in acetate metabolism, were downregulated at 0 and 12 h compared with that at -12 h. Besides, the expression levels of *PsbO*, *NADP-MDH*, and *VPS34* genes, involved in photosynthesis, CO_2_ fixation, and autophagy, were upregulated at 0 and 12 h. Furthermore, the expression levels of some lutein biosynthesis-related genes, including *PDS*, *ZDS*, *LCYB*, and *CYP97C*, were upregulated at 0 h but downregulated at 12 h; however, the expression level of *CYP97A* gene was upregulated at both 0 and 12 h. The expression pattern of the abovementioned genes was consistent with that of RNA-seq data. Hence, the RNA-seq data are reliable and accurate.

## Conclusions

*C. sorokiniana FZU60* grew rapidly at the mixotrophy stage, while lutein accumulation enhanced at the photoautotrophy stage. Based on the physiological, biochemical, and transcriptomic data, the decrease in cell growth after the shift to photoautotrophy could be due to the suppression of glyoxylate cycle and TCA cycle. Besides, the increase in photosynthesis and CO_2_ fixation at the photoautotrophy stage could provide more precursor for lutein accumulation. Moreover, the enhancement of autophagy indicated that ROS level might increase, which could induce lutein biosynthesis simultaneously. Hence, the increase in photosynthesis, CO_2_ fixation, and ROS level after the shift from mixotrophy to photoautotrophy could trigger lutein biosynthesis (Fig. 7).

## Methods

### Microalgal strain and culture conditions

*C. sorokiniana* FZU60 is a newly isolated microalga with high lutein content [9]. The microalgal strain was preserved in a 1.5% (w/v) agar plate with BG11 medium [47].

For pre-culture, microalgal cells were inoculated into a 1-L photobioreactor with BG11 medium at a working volume of 1 L. The microalgal culture was deposited in a light incubator under the conditions of initial pH 7.5, temperature 28 °C, light intensity 250 μmol/m^2^/s, and stirring speed 300 r/min. Besides, 2.5% CO_2_ was constantly aerated into the microalgal culture. The cultivation lasted 3 days.

The pre-cultured microalgal cells were centrifugated at 5000 r/min, and then inoculated into a 1-L photobioreactor with a working volume of 1 L. The microalgal cells were cultivated with a modified BG11 medium (1 g/L NaNO_3_) adding 3 g/L CH_3_COONa with an inoculation size of 100 mg/L. The change in initial NaNO_3_ concentration to 1 g/L is due to that the cell growth and lutein accumulation are better at this concentration under mixotrophic cultivation, and nitrogen is still replete after the shift to photoautotrophy, according to our previous study [9]. The cultivation conditions were similar to that of pre-culture except that the temperature was set at 33 °C, which is due to that both cell growth and lutein accumulation are optimal at this temperature [48]. The culture time at the onset of acetate depletion was denoted as 0 h; thus, microalgal cells were cultivated under mixotrophic and photoautotrophic conditions before and after 0 h, respectively. The microalgal culture was collected at set time intervals to analyze biomass concentration, lutein content, chlorophyll fluorescence parameters, biochemical composition, and transcriptome.

### Determination of biomass concentration

The optical density of 682 nm (OD_682_) of microalgal culture was measured by a spectrophotometer (U-2001, Hitachi, Tokyo, Japan). The biomass concentration of microalgal culture was determined by the equation as follows:

$$y = \, 0.{244}0x + \, 0.0{156 }\left( {R^2 = \, 0.{9961}} \right)$$

where *y* is biomass concentration, and *x* is OD_682_.

### Determination of acetate and nitrogen concentrations

The microalgal culture was sampled every 12 h and filtered through a 0.22 μm filter. The supernatant was collected and properly diluted to determine the acetate and nitrogen concentrations. The acetate concentration was measured by a total organic carbon analyzer (TOC-L CPH, Shimadzu, Kyoto, Japan), as previously reported [9]. The nitrate concentration was analyzed using a colorimetric method [12].

### Chlorophyll fluorescence analysis

The chlorophyll fluorescence of microalgal cells was determined every 12 h from -24 to 36 h and every 24 h from 36 to 72 h. Microalgal culture of 3 mL was sampled in a 5-mL quartz cuvette and kept in the dark for 20 min to reopen PSII reaction centers and relax non-photochemical quenching [19]. The maximum PSII photochemical quantum yield (Fv/Fm) and non-photochemical quenching (NPQ) were determined by a fluorometer (WATER-ED, EDEE0300, Walz, Effeltrich, Germany).

### Analysis of biochemical compositions of microalgal cells

The biochemical compositions of microalgal cells were measured at -12, 0, 12, and 24 h. Besides, lutein content was also measured at 48 and 72 h to investigate the changing trend. The determination of carotenoid, chlorophyll, carbohydrate, and fatty acid contents was carried out according to a previous report [49]. A protein extraction kit (BB-3131-1, BestBio, Shanghai, China) was used to extract protein. The protein content was measured by a Pierce^®^ BCA protein assay kit (Thermo Scientific, Waltham, MA, USA).

### RNA sequencing (RNA-seq)

The microalgal culture was sampled at − 12, 0, and 12 h with three biological replicates for RNA extraction, when acetate was replete (designated as F-12 h group), at the onset of depletion (designated as F0h group), and completely depleted (designated as F12 group), respectively. RNA extraction was carried out with a Trizol reagent kit (Invitrogen, Carlsbad, CA, USA), and the RNA quality was examined on an Agilent 2100 bioanalyzer (Agilent Technologies, Palo Alto, CA, USA). The oligo(dT) beads were used to enrich mRNA, which was then fragmented, and reverse transcribed into cDNA with random primers. Subsequently, a QiaQuick PCR extraction kit (Qiagen, Venlo, The Netherlands) was used to purify the cDNA fragments, which were then end repaired, A base added, and linked to Illumina sequencing adapters. Sequencing was carried out by Gene Denovo Biotechnology Co. (Guangzhou, China) utilizing Illumina novaseq 6000.

### Sequence assembly and annotation

The raw reads were filtered by fastp (version 0.18.0), and the reads containing adapters, ploy-N, and more than 50% of low-quality bases were removed. The reads were then assembled using Trinity software, and the assembly integrity was assessed by BUSCO. The Unigene sequences were then compared to the protein databases NR, SwissProt, KEGG, and COG/KOG by blastx to obtain the protein with the highest sequence similarity, thus achieving the annotation information of the protein function of Unigene.

### Analysis of differentially expressed genes (DEGs)

The DEGs between two distinct groups were analyzed by DESeq2 software [50] and by edgeR [51] between two samples. The genes with the parameters of false discovery rate (FDR) < 0.05 and absolute fold change (FC) ≥ 2 were considered DEGs [52]. The RNA-seq data are shown as log_2_ (FC). The data of log_2_ (FC) and FDR for the genes analyzed in this study are listed in Additional file 2: Table S1–S4.

### Quantitative real-time PCR (qPCR) for validating the expression of selected genes

A total of 10 genes were selected for expression validation, including *ACS* responsible for converting acetate into acetyl-CoA, *ACO* presenting in both the glyoxylate cycle and TCA cycle, *PsbO* presenting in photosynthetic apparatus, *NADP-MDH* involved in CO_2_ fixation, *VPS34* involved in autophagy, and *PDS*, *ZDS*, *LCYB*, *CYP97A*, and *CYP97C* involved in lutein biosynthesis. Total RNA of 1 μg was used for cDNA synthesis using EasyScript® First-Strand cDNA Synthesis SuperMix (TransGen Biotech, Beijing, China). The qPCR was carried out by CFX Connect™ Real-Time PCR Detection System (BIO-RAD, Hercules, CA, USA) with SYBR® Premix Ex TaqTM II (TaKaRa, Japan). The program was as follows: an initial denaturation at 95 °C for 30 s; 40 cycles of denaturation at 95 °C for 5 s and annealing/extension at 60 °C for 20 s; a temperature ramping step for producing melting curve at 60 °C for 15 s. The coding gene of ribosomal protein L19 (RPL19) was used as the reference gene, according to a previous study [53]. The 2^−ΔCt^ method was used to analyze the transcript levels of selected genes based on cycle threshold (Ct) values. All primers are listed in Additional file 2: Table S5.

### Statistical analysis

The data of growth, physiological, and biochemical parameters as well as qPCR analysis are shown as average ± standard deviation. Duncan's test of one-way ANOVA analysis was performed to find significant differences (*p* < 0.05) using IBM SPSS Statistics 24.

## Supplementary Information

**Additional file 1: Figure S1**. The number of unigenes (a) and length distribution of unigenes (b) in *C. sorokiniana* FZU60. **Figure S2**. The numbers of differentially expressed genes among three treatment groups.

**Additional file 2: Table S1** RNA-Seq data for the genes involved in glyoxylate cycle and TCA cycle. **Table S2** RNA-Seq data for the genes involved in photosynthesis and CO_2_ fixation. **Table S3** RNA-Seq data for the genes involved in autophagy. **Table S4** RNA-Seq data for the genes involved in carotenoid biosynthesis. **Table S5** Primers used for expression validation of selected genes by qRT-PCR.

## Data Availability

All data generated or analyzed during this study are included in this manuscript and its supplementary information files. The raw data of RNA-seq generated in this study have been deposited in the Genome Sequence Archive (accession number: CRA008480) in BIG Data Center (http://bigd.big.ac.cn), Beijing Institute of Genomics (BIG), China Academy of Sciences.

## Abbreviations

Cyt *b*_6_*f*: Cytochrome *b*_6_*f*
DEGs: Differentially expressed genes
F-12 h: Samples collected at 12 h before acetate depletion
F0h: Samples collected at the time point of the onset of acetate depletion
F12: Samples collected at 12 h after acetate depletion
FC: Fold change
FDR: False discovery rate
Fv/Fm: The maximum PSII photochemical quantum yield
LHCs: Light-harvesting complexes
NPQ: Non-photochemical quenching
qPCR: Quantitative real-time PCR
RNA-seq: RNA sequencing
ROS: Reactive oxygen species
PSII: Photosystem II
PSI: Photosystem I
