# Supplementary material for: Unveiling the underlying molecular mechanisms of high lutein production efficiency in Chlorella sorokiniana FZU60 under a mixotrophy/photoautotrophy two-stage strategy by transcriptomic, physiological, and biochemical analyses
Source: Biotechnol Biofuels Bioprod. 2023 Mar 15;16:47. doi: 10.1186/s13068-023-02300-8 (PMC10018854; doi:10.1186/s13068-023-02300-8)
Supplement: Supplementary file 1 — Additional file 1: Figure S1. The number of unigenes (a) and length distribution of unigenes (b) in C. sorokiniana FZU60. Figure S2. The numbers of differentially expressed genes among three treatment groups. [file 13068_2023_2300_MOESM1_ESM.docx]

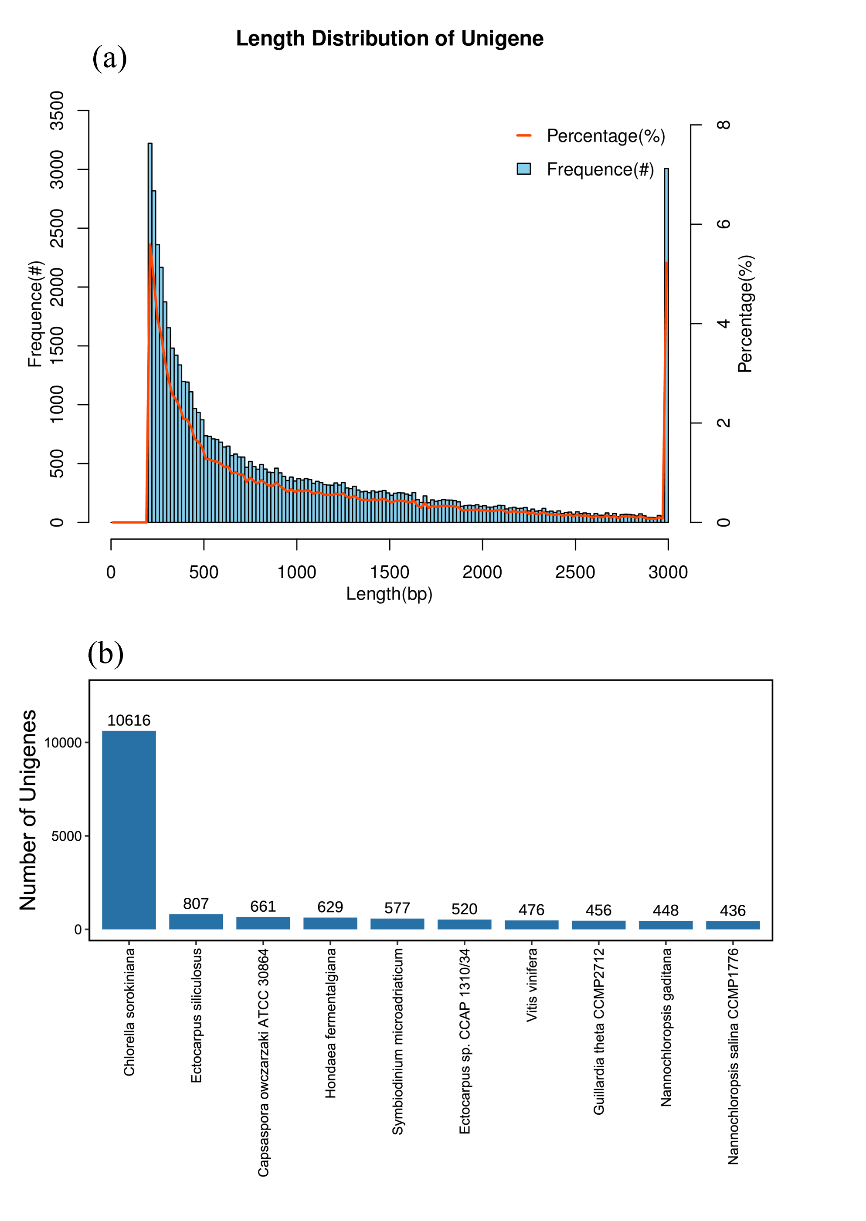


Figure S1. The number of unigenes (a) and length distribution of unigenes (b) in *C. sorokiniana* FZU60.


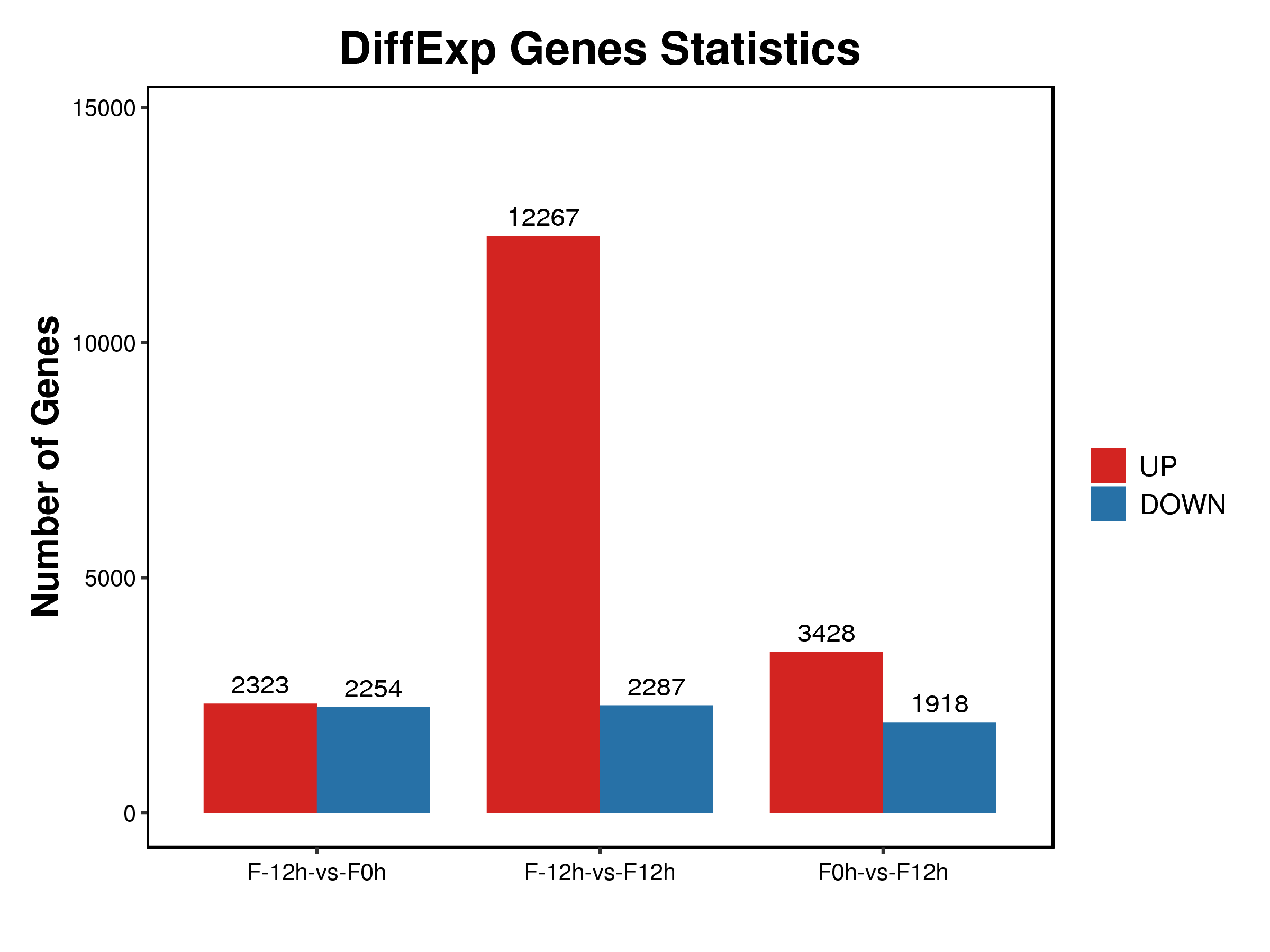


Figure S2. The numbers of differentially expressed genes among three treatment groups.
