# Supplementary material for: Unveiling the underlying molecular mechanisms of high lutein production efficiency in Chlorella sorokiniana FZU60 under a mixotrophy/photoautotrophy two-stage strategy by transcriptomic, physiological, and biochemical analyses
Source: Biotechnol Biofuels Bioprod. 2023 Mar 15;16:47. doi: 10.1186/s13068-023-02300-8 (PMC10018854; doi:10.1186/s13068-023-02300-8)
Supplement: Supplementary file 2 — Additional file 2: Table S1 RNA-Seq data for the genes involved in glyoxylate cycle and TCA cycle. Table S2 RNA-Seq data for the genes involved in photosynthesis and CO2 fixation. Table S3 RNA-Seq data for the genes involved in autophagy. Table S4 RNA-Seq data for the genes involved in carotenoid biosynthesis. Table S5 Primers used for expression validation of selected genes by qRT-PCR. [file 13068_2023_2300_MOESM2_ESM.docx]

Table S1 RNA-Seq data for the genes involved in glyoxylate cycle and TCA cycle.

| Gene ID | Annotation | Abbreviation | F-12h vs F0h | | F-12h vs F12h | |
| --- | --- | --- | --- | --- | --- | --- |
|  |  |  | Log_2_(FC) | FDR | Log_2_(FC) | FDR |
| Unigene0047742 | acetyl-CoA synthetase | ACS | -10.146 | 3.13E-05 | 0.775 | 0.929221082 |
| Unigene0013552 | acetyl-CoA synthetase | ACS | -5.136 | 1.73E-24 | -2.706 | 3.80E-14 |
| Unigene0004232 | citrate synthase | CS | -5.446 | 5.72E-108 | -6.007 | 2.25E-87 |
| Unigene0004231 | citrate synthase | CS | 3.817 | 3.61E-06 | 4.608 | 1.52E-07 |
| Unigene0049358 | aconitate hydratase | ACO | -7.857 | 9.34E-187 | -4.656 | 4.18E-62 |
| Unigene0025728 | isocitrate lyase | ICL | 0.949 | 0.957016657 | 4.008 | 1.11E-05 |
| Unigene0049866 | isocitrate lyase | ICL | 2.707 | 0.539879293 | 6.746 | 7.93E-08 |
| Unigene0047148 | malate synthase | MLS | -6.274 | 3.22E-54 | -5.196 | 7.64E-61 |
| Unigene0044272 | isocitrate dehydrogenase | IDH | -1.329 | 1.18E-12 | -1.860 | 4.41E-12 |
| Unigene0035873 | isocitrate dehydrogenase | IDH | -3.374 | 6.24E-45 | -3.456 | 9.54E-27 |
| Unigene0013457 | 2-oxoglutarate dehydrogenase | OGDH | -4.588 | 1.44E-74 | -3.109 | 2.24E-28 |
| Unigene0034054 | dihydrolipoamide succinyltransferase | DLST | -4.511 | 5.07E-102 | -3.187 | 5.04E-28 |
| Unigene0054638 | dihydrolipoamide succinyltransferase | DLST | -4.336 | 4.11E-68 | -3.115 | 2.18E-20 |
| Unigene0010908 | succinyl-CoA synthetase | LSC | -7.691 | 3.15E-207 | -3.934 | 1.79E-39 |
| Unigene0038154 | succinyl-CoA synthetase | LSC | -7.647 | 1.76E-221 | -3.986 | 4.07E-44 |
| Unigene0005840 | succinate dehydrogenase | SDH | -10.276 | 0.000606006 | -10.276 | 0.000161255 |
| Unigene0015710 | succinate dehydrogenase | SDH | -8.503 | 6.80E-226 | -4.302 | 1.79E-41 |
| Unigene0005841 | succinate dehydrogenase | SDH | -8.089 | 1.33E-247 | -3.688 | 1.36E-30 |
| Unigene0028046 | succinate dehydrogenase | SDH | 2.364 | 0.028568552 | 3.737 | 8.68E-06 |
| Unigene0052810 | fumarate hydratase | FUM | -2.710 | 3.46E-43 | -3.235 | 1.23E-34 |
| Unigene0033292 | malate dehydrogenase | MDH | -9.975 | 0.000534181 | -5.431 | 0.000680274 |
| Unigene0033289 | malate dehydrogenase | MDH | -7.395 | 2.01E-76 | -4.049 | 4.98E-60 |

Table S2 RNA-Seq data for the genes involved in photosynthesis and CO_2_ fixation.

| Gene ID | Annotation | Abbreviation | F-12h vs F0h | | F-12h vs F12h | |
| --- | --- | --- | --- | --- | --- | --- |
|  |  |  | Log_2_(FC) | FDR | Log_2_(FC) | FDR |
| Unigene0047650 | photosystem II oxygen-evolving enhancer protein 1 | PsbO | 4.708 | 1.98E-21 | 3.838 | 1.08E-07 |
| Unigene0028062 | cytochrome b6f complex iron-sulfur subunit | PetC | 4.574 | 0.000112556 | 3.491 | 0.035255181 |
| Unigene0054807 | cytochrome b6f complex iron-sulfur subunit | PetC | 1.182 | 0.041997057 | 0.314 | 0.18197658 |
| Unigene0013097 | F-type H^+^-transporting ATPase subunit gamma | gamma | 1.376 | 0.00032381 | 0.927 | 0.973169951 |
| Unigene0012253 | F-type H+-transporting ATPase subunit delta | delta | 1.231 | 0.64878692 | 2.811 | 0.008791615 |
| Unigene0054180 | F-type H^+^-transporting ATPase subunit a | a | 3.576 | 0.007080152 | 4.030 | 0.004693049 |
| Unigene0010498 | light-harvesting complex I chlorophyll a/b binding protein 1 | Lhca1 | 8.163 | 0.823212568 | 10.613 | 0.01737936 |
| Unigene0040422 | light-harvesting complex I chlorophyll a/b binding protein 1 | Lhca1 | 3.426 | 0.000747384 | -0.337 | 0.189984019 |
| Unigene0006129 | light-harvesting complex I chlorophyll a/b binding protein 2 | Lhca2 | -1.168 | 1.32E-05 | -1.166 | 5.01E-05 |
| Unigene0032698 | light-harvesting complex II chlorophyll a/b binding protein 1 | Lhcb1 | 1.264 | 0.01630261 | 1.101 | 0.759642819 |
| Unigene0038370 | light-harvesting complex II chlorophyll a/b binding protein 1 | Lhcb1 | -3.035 | 0.001959857 | -3.695 | 0.000103844 |
| Unigene0055288 | light-harvesting complex II chlorophyll a/b binding protein 2 | Lhcb2 | 1.367 | 0.006126564 | 1.149 | 0.701097113 |
| Unigene0036955 | ribulose-bisphosphate carboxylase | RBSC | 2.244 | 3.08E-05 | 1.406 | 0.250298108 |
| Unigene0054643 | phosphoglycerate kinase | PGK | -3.433 | 9.78E-63 | -2.592 | 1.01E-27 |
| Unigene0054644 | phosphoglycerate kinase | PGK | 2.188 | 2.59E-09 | 0.972 | 0.82385199 |
| Unigene0038306 | glyceraldehyde 3-phosphate dehydrogenase | GAPDH | -9.074 | 6.43E-172 | -3.518 | 1.40E-41 |
| Unigene0039050 | glyceraldehyde 3-phosphate dehydrogenase | GAPDH | -7.371 | 6.68E-137 | -4.328 | 6.09E-57 |
| Unigene0041201 | glyceraldehyde 3-phosphate dehydrogenase | GAPDH | 2.156 | 9.39E-05 | 4.638 | 1.50E-09 |
| Unigene0002774 | glyceraldehyde 3-phosphate dehydrogenase | GAPDH | 5.063 | 9.21E-25 | 3.750 | 9.14E-08 |
| Unigene0036901 | fructose-bisphosphate aldolase | FBA | -5.180 | 9.63E-55 | -2.716 | 1.23E-29 |
| Unigene0026692 | fructose-bisphosphate aldolase | FBA | 1.690 | 0.000133852 | -0.580 | 0.001216752 |
| Unigene0009908 | fructose-bisphosphate aldolase | FBA | 1.762 | 0.006167975 | 4.360 | 6.41E-12 |
| Unigene0031676 | fructose-bisphosphate aldolase | FBA | 3.039 | 1.10E-17 | 1.182 | 0.656299729 |
| Unigene0050989 | fructose-1,6-bisphosphatase I | FBP | -4.208 | 7.07E-97 | -3.556 | 4.29E-40 |
| Unigene0038377 | fructose-1,6-bisphosphatase I | FBP | 2.309 | 5.83E-12 | 0.887 | 0.991292739 |
| Unigene0032785 | transketolase | TKL | 2.580 | 1.78E-16 | 1.652 | 0.125297089 |
| Unigene0016014 | sedoheptulose-1,7-bisphosphatase | SBP | 2.788 | 1.52E-17 | 1.655 | 0.094924439 |
| Unigene0056283 | ribulose-phosphate 3-epimerase | RPE | 2.851 | 1.14E-18 | 1.237 | 0.539040443 |
| Unigene0042993 | ribose 5-phosphate isomerase | RPI | 1.750 | 1.11E-07 | -1.114 | 8.18E-08 |
| Unigene0012996 | phosphoribulokinase | PRK | 2.588 | 4.91E-15 | 1.179 | 0.601541405 |
| Unigene0025251 | phosphoenolpyruvate carboxylase | PPC | 1.832 | 0.000111316 | 0.285 | 0.175193043 |
| Unigene0056535 | phosphoenolpyruvate carboxylase | PPC | 1.975 | 0.018577813 | -0.797 | 0.00600698 |
| Unigene0048147 | malate dehydrogenase | NADP-MDH | 3.685 | 3.03E-59 | 3.321 | 2.02E-13 |
| Unigene0000110 | pyruvate orthophosphate dikinase | PPDK | -5.841 | 6.12E-90 | -1.884 | 2.00E-19 |
| Unigene0056322 | malic enzyme | ME | -2.090 | 2.97E-23 | -1.734 | 1.61E-11 |
| Unigene0025846 | malic enzyme | ME | -1.602 | 2.51E-08 | -1.415 | 1.78E-11 |
| Unigene0056321 | malic enzyme | ME | 3.886 | 3.92E-16 | 1.849 | 0.10268952 |

Table S3 RNA-Seq data for the genes involved in autophagy.

| Gene ID | Annotation | Abbreviation | F-12h vs F0h | | F-12h vs F12h | |
| --- | --- | --- | --- | --- | --- | --- |
|  |  |  | Log_2_(FC) | FDR | Log_2_(FC) | FDR |
| Unigene0050853 | autophagy-related protein 1 | ATG1 | 1.482 | 0.885870327 | 3.832 | 0.002871317 |
| Unigene0046800 | autophagy-related protein 1 | ATG1 | 1.428 | 0.589271435 | 4.014 | 5.26E-06 |
| Unigene0003831 | autophagy-related protein 1 | ATG1 | 2.105 | 0.290968308 | 4.084 | 0.000663668 |
| Unigene0035595 | autophagy-related protein 1 | ATG1 | 1.952 | 0.849020747 | 4.065 | 0.009981096 |
| Unigene0004556 | autophagy-related protein 1 | ATG1 | 1.382 | 0.855942191 | 4.495 | 7.35E-06 |
| Unigene0040314 | autophagy-related protein 1 | ATG1 | 2.511 | 0.47264926 | 5.728 | 2.65E-06 |
| Unigene0036082 | autophagy-related protein 1 | ATG1 | 2.959 | 0.338078818 | 5.991 | 4.01E-06 |
| Unigene0013238 | autophagy-related protein 1 | ATG1 | 2.503 | 0.524838727 | 6.605 | 1.34E-10 |
| Unigene0037310 | autophagy-related protein 1 | ATG1 | 4.544 | 0.974820097 | 9.922 | 0.036484896 |
| Unigene0049214 | autophagy-related protein 1 | ATG1 | 7.129 | 0.637238548 | 10.576 | 0.001501202 |
| Unigene0050828 | autophagy-related protein 9 | ATG9 | 1.528 | 0.006126564 | 1.379 | 0.359897018 |
| Unigene0041394 | autophagy-related protein 18 | ATG18 | 1.340 | 0.960255869 | 4.053 | 0.018443589 |
| Unigene0041833 | autophagy-related protein 6 | ATG6 | 2.322 | 0.734188809 | 4.934 | 0.002452016 |
| Unigene0012175 | phosphoinositide-3-kinase | VPS15 | 1.562 | 0.948414411 | 4.063 | 0.022588021 |
| Unigene0027206 | phosphoinositide-3-kinase | VPS15 | 2.288 | 0.554199045 | 5.544 | 3.80E-06 |
| Unigene0023752 | phosphatidylinositol 3-kinase | VPS34 | 0.170 | 0.974820097 | 5.185 | 0.025474609 |
| Unigene0056425 | phosphatidylinositol 3-kinase | VPS34 | 2.019 | 0.657782173 | 5.505 | 1.34E-06 |
| Unigene0001995 | autophagy-related protein 3 | ATG3 | 1.264 | 0.885674067 | 3.970 | 1.50E-05 |
| Unigene0056751 | autophagy-related protein 4 | ATG4 | 1.886 | 0.591430908 | 5.785 | 1.01E-09 |
| Unigene0036524 | autophagy-related protein 4 | ATG4 | 2.585 | 0.934214637 | 6.119 | 0.006855416 |
| Unigene0006651 | autophagy-related protein 4 | ATG4 | 3.700 | 0.675136801 | 6.728 | 0.002286968 |
| Unigene0039194 | autophagy-related protein 4 | ATG4 | 4.755 | 0.272517885 | 8.248 | 9.94E-06 |
| Unigene0008433 | autophagy-related protein 4 | ATG4 | 6.644 | 0.755160676 | 10.697 | 1.59E-05 |
| Unigene0054831 | autophagy-related protein 7 | ATG7 | 1.550 | 0.93177246 | 4.714 | 0.000335191 |
| Unigene0056104 | autophagy-related protein 8 | ATG8 | 2.853 | 2.21E-16 | 2.449 | 0.000605863 |
| Unigene0044387 | autophagy-related protein 8 | ATG8 | 2.995 | 0.034504071 | 6.059 | 3.60E-12 |
| Unigene0056654 | autophagy-related protein 8 | ATG8 | 2.603 | 0.158135847 | 5.134 | 9.61E-07 |
| Unigene0003842 | autophagy-related protein 8 | ATG8 | 2.591 | 0.480029385 | 5.419 | 1.78E-05 |
| Unigene0055070 | autophagy-related protein 8 | ATG8 | 2.338 | 0.865274711 | 5.481 | 0.001449785 |
| Unigene0030832 | autophagy-related protein 8 | ATG8 | 8.276 | 0.883837675 | 10.607 | 0.025427884 |
| Unigene0056167 | autophagy-related protein 10 | ATG10 | 2.009 | 0.000856178 | 0.937 | 0.973169951 |

Table S4 RNA-Seq data for the genes involved in carotenoid biosynthesis.

| Gene ID | Annotation | Abbreviation | F-12h vs F0h | | F-12h vs F12h | |
| --- | --- | --- | --- | --- | --- | --- |
|  |  |  | Log_2_(FC) | FDR | Log_2_(FC) | FDR |
| Unigene0041231 | phytoene desaturase | PDS | 1.590 | 0.000189443 | -0.937 | 5.04E-07 |
| Unigene0024163 | zeta-carotene desaturase | ZDS | 1.781 | 2.04E-05 | -0.952 | 3.16E-07 |
| Unigene0046884 | lycopene beta-cyclase | LCYB | 1.526 | 0.000194897 | -1.198 | 4.29E-08 |
| Unigene0029479 | cytochrome P450 carotenoid hydroxylase A | CYP97A | 1.202 | 0.974820097 | 5.256 | 0.004022262 |
| Unigene0041884 | cytochrome P450 carotenoid hydroxylase A | CYP97A | 1.999 | 4.71E-07 | 0.632 | 0.595647323 |
| Unigene0012343 | cytochrome P450 carotenoid hydroxylase C; | CYP97C | 6.322 | 0.857937182 | 10.665 | 2.17E-05 |
| Unigene0019746 | cytochrome P450 carotenoid hydroxylase C; | CYP97C | 1.006 | 0.780232981 | -2.383 | 4.32E-14 |

Table S5 Primers used for expression validation of selected genes by qRT-PCR.

| Primer name | Forward | Reverse |
| --- | --- | --- |
| ACS | GGACTCGGAGGATACCCTGTT | TCACGTAGGAATGGCCGGT |
| ACO | TGGTGCTGATCAAGGCCAAG | CCGTCAGCTGGTTCTTCACA |
| PsbO | AGCGAGTTTGAGAAGTCCACC | TGAACATGAAGGGCACCATCTC |
| NADP-MDH | TGACGAGGACCTGGTGTTCT | TCTACAGCCAGCCCAATGC |
| VPS34 | GAGAGTTTCCCTCTCGGAAGC | TCGGTGATTAACCCGGGAGT |
| PDS | GGTGGAGAAGACGTTCCAGG | GACTCCAGCACAATCGGCTT |
| ZDS | CTCGCCTGTCGACTACTACAAG | CACTTGAGGTTGAGGTCCTTGG |
| LCYB | AAGATCTTCCTGGAGGAGACGAG | CTCCTCAATGCTCAGCACCTTG |
| CYP97A | GGGAGATACTTGGAAATCACGCA | CGCCCAACAACTTCTCCAGT |
| CYP97C | AGAAGATCGACACCGGCAAG | GCTCTCCAGCATCCACTTGT |
| RPL19 | GTCTGGCTGGACCCCAATGA | GCCTCTGCTGCGGTGC |
